# Genome sequence of the thermophilic fresh-water bacterium *Spirochaeta caldaria* type strain (H1^T^), reclassification of *Spirochaeta caldaria*, *Spirochaeta stenostrepta,* and *Spirochaeta zuelzerae* in the genus *Treponema* as *Treponema caldaria* comb. nov., *Treponema stenostrepta* comb. nov., and *Treponema zuelzerae* comb. nov., and emendation of the genus *Treponema*

**DOI:** 10.4056/sigs.3096473

**Published:** 2013-04-15

**Authors:** Birte Abt, Markus Göker, Carmen Scheuner, Cliff Han, Megan Lu, Monica Misra, Alla Lapidus, Matt Nolan, Susan Lucas, Nancy Hammon, Shweta Deshpande, Jan-Fang Cheng, Roxanne Tapia, Lynne A. Goodwin, Sam Pitluck, Konstantinos Liolios, Ioanna Pagani, Natalia Ivanova, Konstantinos Mavromatis, Natalia Mikhailova, Marcel Huntemann, Amrita Pati, Amy Chen, Krishna Palaniappan, Miriam Land, Loren Hauser, Cynthia D. Jeffries, Manfred Rohde, Stefan Spring, Sabine Gronow, John C. Detter, James Bristow, Jonathan A. Eisen, Victor Markowitz, Philip Hugenholtz, Nikos C. Kyrpides, Tanja Woyke, Hans-Peter Klenk

**Affiliations:** 1 Leibniz Institute DSMZ - German Collection of Microorganisms and Cell Cultures, Braunschweig, Germany; 2 DOE Joint Genome Institute, Walnut Creek, California, USA; 3 Los Alamos National Laboratory, Bioscience Division, Los Alamos, New Mexico, USA; 4 Biological Data Management and Technology Center, Lawrence Berkeley National Laboratory, Berkeley, California, USA; 5 Oak Ridge National Laboratory, Oak Ridge, Tennessee, USA; 6 HZI – Helmholtz Centre for Infection Research, Braunschweig, Germany; 7 University of California Davis Genome Center, Davis, California, USA; 8 Australian Centre for Ecogenomics, School of Chemistry and Molecular Biosciences, The University of Queensland, Brisbane, Australia

**Keywords:** obligately anaerobic, thermophilic, spiral-shaped, motile, periplasmic flagella, Gram-negative, chemoorganotrophic, *Spirochaetaceae*, *Spirochaeta*, *Treponema*, GEBA

## Abstract

*Spirochaeta caldaria* Pohlschroeder *et al*. 1995 is an obligately anaerobic, spiral-shaped bacterium that is motile *via* periplasmic flagella. The type strain, H1^T^, was isolated in 1990 from cyanobacterial mat samples collected at a freshwater hot spring in Oregon, USA, and is of interest because it enhances the degradation of cellulose when grown in co-culture with *Clostridium thermocellum*. Here we provide a taxonomic re-evaluation for *S. caldaria* based on phylogenetic analyses of 16S rRNA sequences and whole genomes, and propose the reclassification of *S. caldaria* and two other *Spirochaeta* species as members of the emended genus *Treponema.* Whereas genera such as *Borrelia* and *Sphaerochaeta* possess well-distinguished genomic features related to their divergent lifestyles, the physiological and functional genomic characteristics of *Spirochaeta* and *Treponema* appear to be intermixed and are of little taxonomic value. The 3,239,340 bp long genome of strain H1^T^ with its 2,869 protein-coding and 59 RNA genes is a part of the *** G****enomic* ***E****ncyclopedia of* ***Bacteria**** and* ***Archaea***** project.

## Introduction

Strain H1^T^ (= DSM 7334 = ATCC 51460) is the type strain of the species *Spirochaeta caldaria* [1,2] in the genus *Spirochaeta* (which currently contains 19 validly named species [3,4]) and was first isolated from cyanobacterial mat samples collected at a freshwater hot spring in Oregon, USA [1]. The genus name was derived from the latinized Greek words 'speira' meaning 'a coil' and 'chaitê' meaning 'hair', yielding the Neo-Latin 'Spirochaeta', 'coiled hair' [3]. The species epithet is derived from the Latin adjective 'caldaria', 'pertaining to warm water' (intended to mean inhabiting warm water) [3]. References to *S. caldaria* in PubMed are rather sparse. In 1996 Paster *et al*. reported *S. caldaria* as the closest relative to a spirochaete clone from the hindguts of an African higher termite, *Nasutermites lujae* [5], an observation that was underlined three years later when Lilburn *et al*. identified *S. caldaria* as a close relative of the majority of the ‘spirochaetes’ in the gut of the termite *Reticulitermes flavipes* [6]. In the same year (1999) Ohkuma *et al.* confirmed this observation for symbiotic ‘spirochaetes’ in the gut of diverse termites [7]. Here we present a summary classification and a set of features for *S. caldaria* strain H1^T^, together with the description of the complete genome sequencing and annotation.

## Features of the organism

A representative genomic 16S rRNA sequence of *S. caldaria* H1^T^ was compared using NCBI BLAST [8,9] under default settings (e.g., considering only the high-scoring segment pairs (HSPs) from the best 250 hits) with the most recent release of the Greengenes database [10] and the relative frequencies of taxa and keywords (reduced to their stem [11]) were determined, weighted by BLAST scores. The most frequently occurring genera were *Spirochaeta* (79.9%) and *Treponema* (20.1%) (17 hits in total). Regarding the two hits to sequences from members of the species, the average identity within HSPs was 99.4%, whereas the average coverage by HSPs was 98.4%. Regarding the five hits to sequences from other members of the genus, the average identity within HSPs was 94.3%, whereas the average coverage by HSPs was 96.3%. Among all other species, the one yielding the highest score was *“Spirochaeta taiwanensis”* AY35103, which corresponded to an identity of 95.2% and an HSP coverage of 94.4%. (Note that the Greengenes database uses the INSDC (= EMBL/NCBI/DDBJ) annotation, which is not an authoritative source for nomenclature or classification.) The highest-scoring environmental sequence was FJ462015 ('Microbial ecology industrial digester mesophilic anaerobic reactor fed effluent chemical industry clone 71a'), which showed an identity of 97.9% and an HSP coverage of 98.1%. The most frequently occurring keywords within the labels of all environmental samples which yielded hits were 'termit' (26.5%), 'hindgut' (17.8%), 'gut' (8.6%), 'homogen' (5.5%) and 'flagel' (2.1%) (233 hits in total), which is in line with previous observations about close relatives in termite guts [5-7]. Environmental samples which yielded hits of a higher score than the highest scoring species were not found.

**Figure 1:**
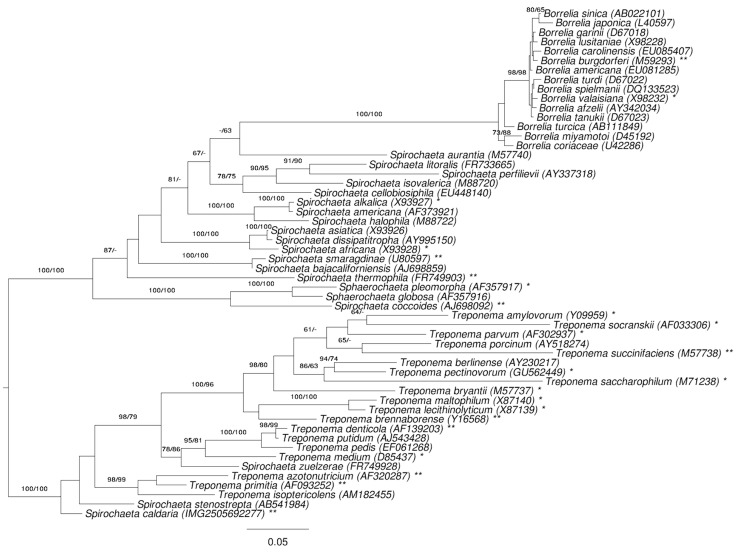
Phylogenetic tree highlighting the position of *S. caldaria* relative to the type strains of the other species within the family *Spirochaetaceae*. The tree was inferred from 1,362 aligned characters [12,13] of the 16S rRNA gene sequence under the maximum likelihood (ML) criterion [14]. Rooting was done initially using the midpoint method [15] and then checked for its agreement with the current classification (Table 1). The branches are scaled in terms of the expected number of substitutions per site. Numbers adjacent to the branches are support values from 1,000 ML bootstrap replicates [16] (left) and from 1,000 maximum-parsimony bootstrap replicates [17] (right) if larger than 60%. Lineages with type strain genome sequencing projects registered in GOLD [18] are labeled with one asterisk, those also listed as 'Complete and Published' with two asterisks [19-23] (for *S. thermophila*, *T. azotonutricium* and *T. primitia* see CP002903, CP001841 and CP001883). Note: *Spirochaeta coccoides* was effectively renamed to *Sphaerochaeta coccoides* in [19] (see Validation List 147 [24].)

Figure 1 shows the phylogenetic neighborhood of *S. caldaria* in a 16S rRNA based tree. The sequences of the three 16S rRNA gene copies in the genome differ from each other by up to three nucleotides, and differ by up to four nucleotides from the previously published 16S rRNA sequence EU580141.

### Morphology and physiology

**Figure 2:**
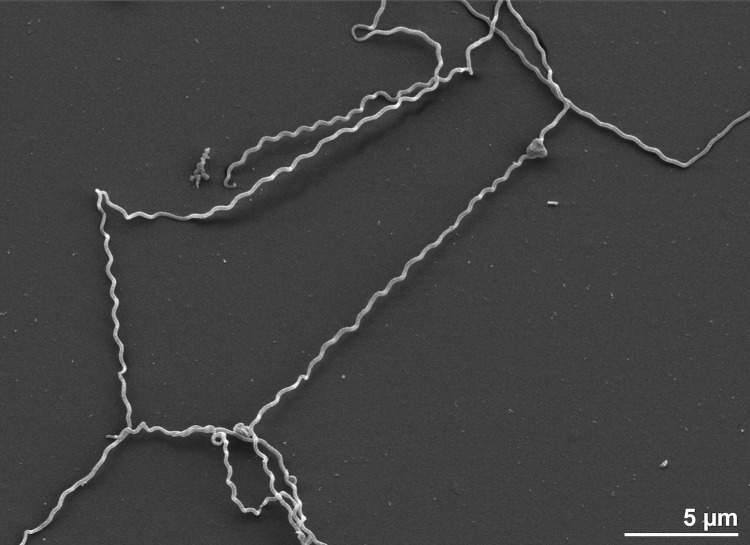
Scanning electron micrograph of *S. caldaria* strain H1^T^

Cells of *S. caldaria* were helical, 0.2 to 0.3 µm in diameter and 15 to 25 µm in length (Figure 2); spherical bodies were seen in stationary-phase cultures (not visible in Figure 2). The cells are motile by two periplasmic flagella in a 1:2:1 arrangement [1]. *S. caldaria* is a Gram-negative, strictly anaerobic, thermophile (Table 1) with an optimal growth temperature between 48°C and 52°C, and no growth observed above 60°C or below 25°C [1]. The pH range for growth is 5.8-8.5, with an optimum at pH 7.2-7.5 [1]. *S. caldaria* tolerates a NaCl concentration of up to 0.25% (wt/vol), but no growth was observed in the presence of 0.4% (wt/vol) NaCl or higher concentrations [1]. On agar plates strain H1^T^ forms white, fluffy, cotton-ball like colonies.

**Table 1:** Classification and general features of *S. caldaria* H1^T^ according to the MIGS recommendations [25] and the NamesforLife database [4].

MIGS ID	Property	Term	Evidence code
	Current classification	Domain *Bacteria*	TAS [26]
		Phylum *Spirochaetae*	TAS [27,28]
		Class *Spirochaetes*	TAS [29,30]
		Order *Spirochaetales*	TAS [31,32]
		Family *Spirochaetaceae*	TAS [30,31,33]
		Genus *Spirochaeta*	TAS [31,34-36]
		Species *Spirochaeta caldaria*	TAS [1,2]
		Type strain H1	TAS [1,2]
	Gram stain	negative	TAS [2]
	Cell shape	spiral shaped	TAS [2]
	Motility	motile	TAS [2]
	Sporulation	none	TAS [2]
	Temperature range	thermophile	TAS [2]
	Optimum temperature	48-52°C	TAS [2]
	Salinity	<0.4%	TAS [2]
MIGS-22	Oxygen requirement	obligately anaerobic	TAS [2]
	Carbon source	carbohydrates	TAS [2]
	Energy metabolism	chemoorganotroph	TAS [2]
MIGS-6	Habitat	fresh water, hot spring	TAS [2]
MIGS-15	Biotic relationship	free living	TAS [2]
MIGS-14	Pathogenicity	none	TAS [2]
	Biosafety level	1	TAS [37]
	Isolation	hot spring	TAS [2]
MIGS-4	Geographic location	Hunter's Hot Spring, Oregon	TAS [2]
MIGS-5	Sample collection time	August 1990	NAS
MIGS-4.1	Latitude	42.222	NAS
MIGS-4.2	Longitude	-120.368	NAS
MIGS-4.3	Depth	not reported	
MIGS-4.4	Altitude	not reported	

Evidence codes - TAS: Traceable Author Statement (i.e., a direct report exists in the literature); NAS: Non-traceable Author Statement (i.e., not directly observed for the living, isolated sample, but based on a generally accepted property for the species, or anecdotal evidence). Evidence codes are from of the Gene Ontology project [75].

*S. caldaria* utilizes pentoses, hexoses and disaccharides as carbon and energy sources. Amino acids cannot be fermented. Glucose is fermented to H_2_, CO_2_, acetate and lactate as the main fermentation products, ethanol is not produced [1]. H1^T^ is able to ferment L-arabinose, D-galactose, D-glucose, D-mannose, D-fructose, D-xylose, cellobiose, cellotriose, cellotetraose, lactose, maltose, sucrose and starch. D-ribose, mannitol, cellulose, xylan, glycerol, peptone, casein hydrolysate, and sodium acetate are not utilized [1]. Exogenous fatty acids, reported to be required by *Treponema* species for cellular lipid synthesis and growth [38], are not required. A supplement with vitamins is, however, required [1]. *S. caldaria* grows in the presence of rifampicin (100 µg/ml of medium), but growth is inhibited by penicillin G, neomycin, chloramphenicol or tetracycline (10 µg/ml of medium each) [1].

## Genome sequencing and annotation

### Genome project history

This organism was selected for sequencing on the basis of its phylogenetic position [39], and is part of the *** G****enomic* ***E****ncyclopedia of* ***Bacteria**** and* ***Archaea***** project [40]. The genome project is deposited in the Genomes On Line Database [18] and the complete genome sequence is deposited in GenBank. Sequencing, finishing and annotation were performed by the DOE Joint Genome Institute (JGI) using state of the art sequencing technology [41]. A summary of the project information is shown in Table 2.

**Table 2:** Genome sequencing project information

**MIGS ID**	**Property**	**Term**
MIGS-31	Finishing quality	Finished
MIGS-28	Libraries used	Three genomic libraries: one 454 pyrosequence standard library, one 454 PE library (12 kb insert size), one Illumina library
MIGS-29	Sequencing platforms	Illumina GAii, 454 GS FLX Titanium
MIGS-31.2	Sequencing coverage	283.3 × Illumina; 26.8 × pyrosequence
MIGS-30	Assemblers	Newbler version 2.3, Velvet 0.7.63, phrap version SPS - 4.24
MIGS-32	Gene calling method	Prodigal
	INSDC ID	CP002868
	GenBank Date of Release	August 12, 2011
	GOLD ID	Gc01874
	NCBI project ID	46527
	Database: IMG-GEBA	2505679006
MIGS-13	Source material identifier	DSM 7334
	Project relevance	Tree of Life, GEBA

### Growth conditions and DNA isolation

*S. caldaria* strain H1^T^, DSM 7334, was grown anaerobically in DSMZ medium 635 (*Spirochaeta caldaria* medium) [42] at 50°C. DNA was isolated from 0.5-1 g of cell paste using MasterPure Gram-positive DNA purification kit (Epicentre MGP04100) following the standard protocol as recommended by the manufacturer with modification st/DL for cell lysis as described in Wu *et al*. 2009 [40]. DNA is available through the DNA Bank Network [43].

### Genome sequencing and assembly

The genome was sequenced using a combination of Illumina and 454 sequencing platforms. All general aspects of library construction and sequencing can be found at the JGI website [44]. Pyrosequencing reads were assembled using the Newbler assembler (Roche). The initial Newbler assembly, consisting of 60 contigs in one scaffold, was converted into a phrap [45] assembly by making fake reads from the consensus, to collect the read pairs in the 454 paired end library. Illumina GAii sequencing data (899.9 Mb) was assembled with Velvet [46] and the consensus sequences were shredded into 2.0 kb overlapped fake reads and assembled together with the 454 data. The 454 draft assembly was based on 121.6 Mb 454 draft data and all of the 454 paired end data. Newbler parameters are -consed -a 50 -l 350 -g -m -ml 20. The Phred/Phrap/Consed software package [45] was used for sequence assembly and quality assessment in the subsequent finishing process. After the shotgun stage, reads were assembled with parallel phrap (High Performance Software, LLC). Possible mis-assemblies were corrected with gapResolution [44], Dupfinisher [46], or sequencing clones bridging PCR fragments with subcloning. Gaps between contigs were closed by editing in Consed, by PCR and by Bubble PCR primer walks (J.-F. Chang, unpublished). A total of 519 additional reactions and 5 shatter libraries were necessary to close gaps and to raise the quality of the finished sequence. Illumina reads were also used to correct potential base errors and increase consensus quality using a software Polisher developed at JGI [47]. The error rate of the completed genome sequence is less than 1 in 100,000. Together, the combination of the Illumina and 454 sequencing platforms provided 310.1 × coverage of the genome. The final assembly contained 285,090 pyrosequence and 24,996,639 Illumina reads.

### Genome annotation

Genes were identified using Prodigal [48] as part of the DOE-JGI genome annotation pipeline [24], followed by a round of manual curation using the JGI GenePRIMP pipeline [49]. The predicted CDSs were translated and used to search the National Center for Biotechnology Information (NCBI) nonredundant database, UniProt, TIGR-Fam, Pfam, PRIAM, KEGG, COG, and InterPro databases. Additional gene prediction analysis and functional annotation was performed within the Integrated Microbial Genomes - Expert Review (IMG-ER) platform [50].

## Genome properties

**Figure 3:**
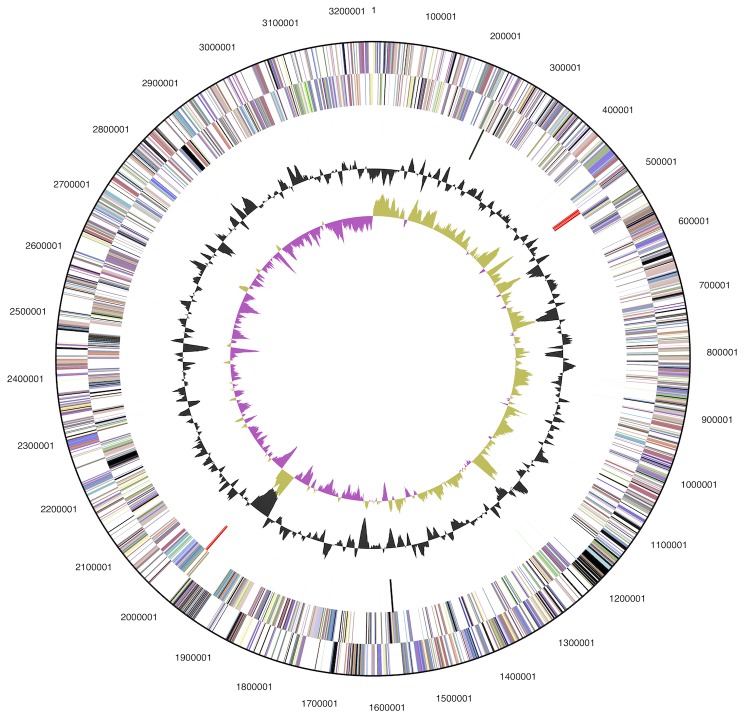
Graphical map of the chromosome. From outside to the center: Genes on forward strand (color by COG categories), Genes on reverse strand (color by COG categories), RNA genes (tRNAs green, rRNAs red, other RNAs black), GC content (black), GC skew (purple/olive).

The genome consists of a 3,239,340 bp long chromosome with a G+C content of 45.6% (Table 3 and Figure 3). Of the 2,928 genes predicted, 2,869 were protein-coding genes, and 59 RNAs; 80 pseudogenes were also identified. The majority of the protein-coding genes (71.0%) were assigned a putative function while the remaining ones were annotated as hypothetical proteins. The distribution of genes into COGs functional categories is presented in Table 4.

**Table 3:** Genome Statistics

**Attribute**	**Value**	**% of Total**
Genome size (bp)	3,239,340	100.00%
DNA coding region (bp)	2,965,950	91.56%
DNA G+C content (bp)	1,476,358	45.58%
Number of replicons	1	
Extrachromosomal elements	0	
Total genes	2,928	100.00%
RNA genes	59	2.02%
rRNA operons	3	
Protein-coding genes	2,869	97.98%
Pseudo genes	80	2.73%
Genes with function prediction	2,078	70.97%
Genes in paralog clusters	1,319	45.05%
Genes assigned to COGs	2,270	77.53%
Genes assigned Pfam domains	2,260	77.19%
Genes with signal peptides	527	18.00%
Genes with transmembrane helices	762	26.02%
CRISPR repeats	0	

**Table 4:** Number of genes associated with the general COG functional categories

**Code**	**Value**	**%age**	**Description**
J	158	6.3	Translation, ribosomal structure and biogenesis
A	0	0.0	RNA processing and modification
K	156	6.2	Transcription
L	125	5.0	Replication, recombination and repair
B	2	0.1	Chromatin structure and dynamics
D	33	1.3	Cell cycle control, cell division, chromosome partitioning
Y	0	0.0	Nuclear structure
V	40	1.6	Defense mechanisms
T	228	9.1	Signal transduction mechanisms
M	142	5.7	Cell wall/membrane/envelope biogenesis
N	86	3.4	Cell motility
Z	0	0.0	Cytoskeleton
W	0	0.0	Extracellular structures
U	50	2.0	Intracellular trafficking, secretion, and vesicular transport
O	91	3.6	Posttranslational modification, protein turnover, chaperones
C	134	5.3	Energy production and conversion
G	296	11.8	Carbohydrate transport and metabolism
E	188	7.5	Amino acid transport and metabolism
F	67	2.7	Nucleotide transport and metabolism
H	77	3.1	Coenzyme transport and metabolism
I	60	2.4	Lipid transport and metabolism
P	77	3.1	Inorganic ion transport and metabolism
Q	26	1.1	Secondary metabolites biosynthesis, transport and catabolism
R	293	11.7	General function prediction only
S	182	7.3	Function unknown
-	658	22.5	Not in COGs

## Insights from the genome sequence, and taxonomic conclusions for *S. caldaria*

### Comparative genomics

**Fig. 4:**
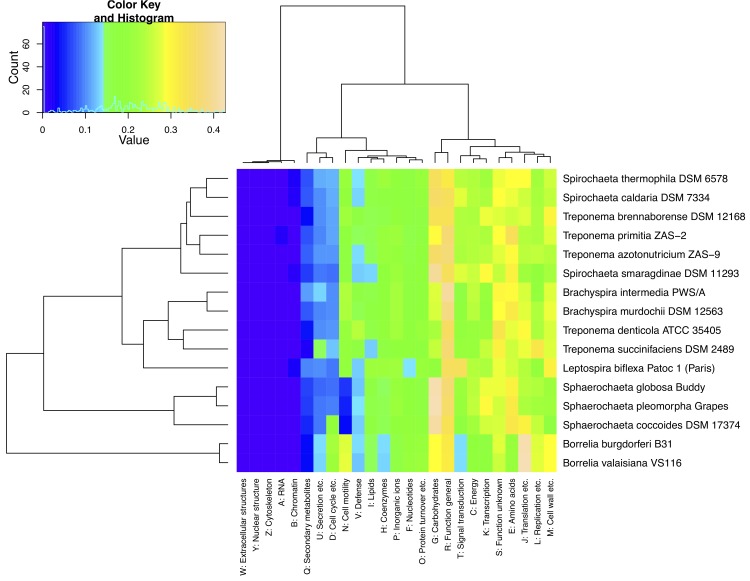
Heatmap showing the distribution of transformed relative COG category counts. The rows represent the genomes, the columns the COG categories. Both rows and columns were rearranged according to their overall (dis-)similarities as represented by the dendrograms on the left and upper side, respectively; for technical details see the opm manual [52].

To assess the composition of the completed *Spirochaetes* type-strain genomes, we extracted the COG IDs from their IMG annotations [50] and determined the absolute and relative numbers of genes present in each COG category [51]. Heatmaps were generated using the opm package [52] for the statistical environment R [53] from the arcsine-square root transformed (see, e.g., p. 386 in [54] for the rationale of this transformation) COG proportions (Fig. 4) and from the log-transformed absolute numbers (data not shown). The results indicate that the relative COG category content mainly reflects changes in life style, with the intracellular parasites (*Borrelia* spp.) and the coccoid forms (*Sphaerochaeta* spp.) forming clusters of their own.

**Fig. 5:**
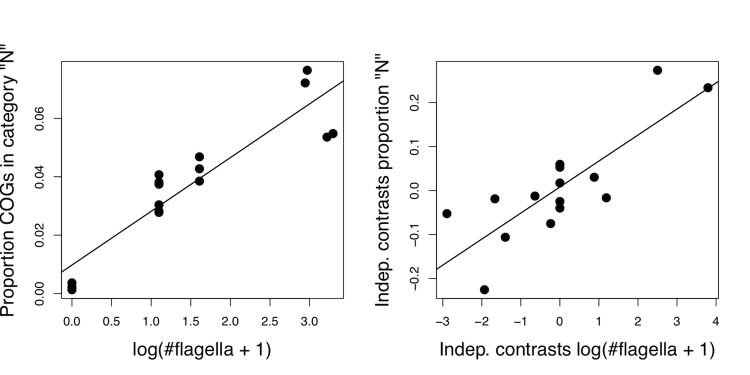
Scatter plots showing the relationship between the number of flagella and the proportion of genes in the COG category N (“Cell motility”). The left picture is based on the uncorrected data, whereas the right graph plots the phylogeny-independent contrast calculated from the numbers used in the left graph. The lines represent the corresponding linear models. For the magnitudes and significances of the correlations, see the main text.

Expectedly, the *Sphaerochaeta* genomes are impoverished with regard to category N (“Cell motility”). The genomes of the flagellated forms, however, also differ regarding their proportion of genes in this category. Hence, we calculated the correlation between this proportion and the average number of flagella reported for each species in the literature [55] (Fig. 5, left side). The correlation was high (0.917) and significant (p < 10^-07^). The number of flagella obviously has a historical component, with flagella lacking in one clade (*Sphaerochaeta*) and the number of flagella being particularly high in other clades (*Borrelia*, *Brachyspira*). To rule out a pseudocorrelation caused by common ancestry (see chapter in [56] for the background), we thus converted the data to phylogeny-independent contrasts using the CONTRASTS program available in the PHYLIP package [57] and the ML tree inferred from the 16S rRNAs from the genome sequences as the underlying phylogeny. The correlation between the contrasts was almost as high (0.818) and significant (p < 10^-05^) (Fig. 5, right side). Thus, *Spirochaetes* appear to rely on increasing their number of motility genes for increasing their number of flagella.

Other relations to the life-style include the lower proportions of many COG categories in the reduced genomes of the *Borrelia* species, apparently as an adaptation to their lifestyle (the higher proportion of genes in category J is simply due to the absolute number of genes in this category being held constant during genome reduction; data not shown), which is considered by some as parasitic, and symbiotic by others. The coccoid forms have an increased proportion of genes in category G, related to carbohydrate transport and metabolism, but this seems not be directly linked to the loss of the typical spirochaete shape, as the *Spirochaeta smaragdinae* genome shows a similarly high proportion of G genes (Fig. 4) and in absolute terms has more genes in this category than *S. coccoides* (data not shown). The coccoid forms have fewer genes in the cell-wall related category M, but this also holds for *S. smaragdinae*.

Further, there seem to be more genes in the defense-related category V in the genomes of the host-associated but non-intracellular genera *Brachyspira* and *Treponema*, but there are exceptions to this rule, such as *T. azotonutricium* (Fig. 4). In contrast to the other genera, neither *Spirochaeta* nor *Treponema* appear as homogeneous genera the COG content of their genomes, even if one considers that *S. caldaria* might be better placed in *Treponema* (see below).

## Taxonomic interpretation for *S. caldaria* and neighboring species in the family *Spirochaetaceae* according to 16S rRNA data

Based on physiological characteristics, the G+C content and the comparison of 16S rRNA sequences, strain H1^T^ was classified into the genus *Spirochaeta* [1]. *S. caldaria* H1^T^ is free living, saccharolytic, obligate anaerobe and possess the ultrastructural features typical of spirochetes. *S. caldaria* differs from all other *Spirochaeta* species, with respect to its thermophilic growth temperature, with the exception of *Spirochaeta thermophila,* which has a temperature optimum between 66 and 68°C [1]. In contrast to the mesophilic *Spirochaeta* species, *S. caldaria* does not produce ethanol as an end-product of D-glucose fermentation [1].

Based on a 16S rRNA sequence comparison, *S. caldaria* as well as *Spirochaeta zuelzerae* and *Spirochaeta stenostrepta* are more closely related to species of *Treponema* (Fig. 1). To rule out the possibility that the discrepancies between 16S rRNA data and taxonomic classification were not caused by either a mix-up or contamination of cultures, we cross-compared the 16S rRNA sequences deposited in INSDC for *S. caldaria* (EU580141 and M71240 in addition to the herein published whole genome sequence), *S. stenostrepta* (AB541984, FR733664, and M88724) and *S. zuelzerae* (FR749928, FR749929 and M88725), respectively. Besides poor sequence quality towards the ends of some sequence deposits, differences between accessions annotated as originating from the same species were not apparent.

The 16S rRNA data and the taxonomic classification of *Spirochaetaceae* are in significant conflict with each other. This problem has already been addressed in detail in one of the previous reports of the GEBA series [19]. The analysis shown in [19] used the classification as phylogenetic constraint, paired-site tests [56] to assess the significance of the differences between the resulting trees, and the ParaFit tests to determine the leaves of the trees that cause these differences [58]. One of the consequences of the earlier study was the assignment of *Spirochaeta coccoides* to *Sphaerochaeta* (compare Fig. 1 with Fig. 5 below). We here focus on our current target species, *S. caldaria*, and comparably problematic taxa.

### Phylogenomic analyses

According to the results from 16S rRNA analysis (Fig. 1) a comparative analysis the genome sequences of *Spirochaeta africana* (GenBank CP003282) and *Treponema primitia* (GenBank CP001843) were performed. The genomes of the sequenced *Spirochaeta* species and *T. primitia* differ significantly in their size. Compared to the genome of *T. primitia* (4.1 Mb, 3,579 protein-coding genes) the genomes of *S. caldaria* (3.2 Mb, 2,928 protein coding genes), and *S. africana* (3.3 Mb, 3,874 protein-coding genes) are smaller in size.

An estimate of the overall similarity among *S. caldaria,* *S. africana* and *T. primitia* was computed with the Genome-to-Genome Distance Calculator (GGDC) [59,60]. This system calculates the distances by comparing the genomes to obtain HSPs (high-scoring segment pairs) and inferring distances from the set of formulas (1, HSP length / total length; 2, identities / HSP length; 3, identities / total length). Table 5 shows the results of the pairwise comparison.

**Table 5:** Pairwise comparison of *S. caldaria* with *S. africana* and *T. primitia* using the GGDC-Genome-to-Genome Distance Calculator.

		**HSP length /** **total length [%]**	**identities /** **HSP length [%]**	**identities /** **total length [%]**
S. caldaria	S. africana	1.62	84.50	1.37
*S. caldaria*	*T. primitia*	6.04	81.92	4.95
*T. primitia*	*S. africana*	1.34	83.99	1.12

The comparison of *S. caldaria* with *T. primitia* yielded the highest scores, 6.04% of the average of genome length are covered with HSPs. The identity within the HSPs was 81.92%, whereas the identity over the whole genome was 4.95%. Lower similarity scores were observed in the comparison of *S. caldaria* with *S. africana* in which only 1.62% of the average of both genome lengths are covered with HSPs. The identity within these HSPs was 84.5%, whereas the identity over the whole genome was only 1.37%.

As expected, those distances relating HSP coverage (formula 1) and number of identical base pairs within HSPs to total genome length (formula 3) are higher between *S. caldaria* and *T. primitia* than between *S. caldaria* and *S. africana*. That the distances relating the number of identical base pairs to total HSP length (formula 2) behave differently indicates that the genomic similarities between *S. caldaria* and *S. africana* are limited to more conserved sequences, a kind of saturation phenomenon [59].

**Figure 6:**
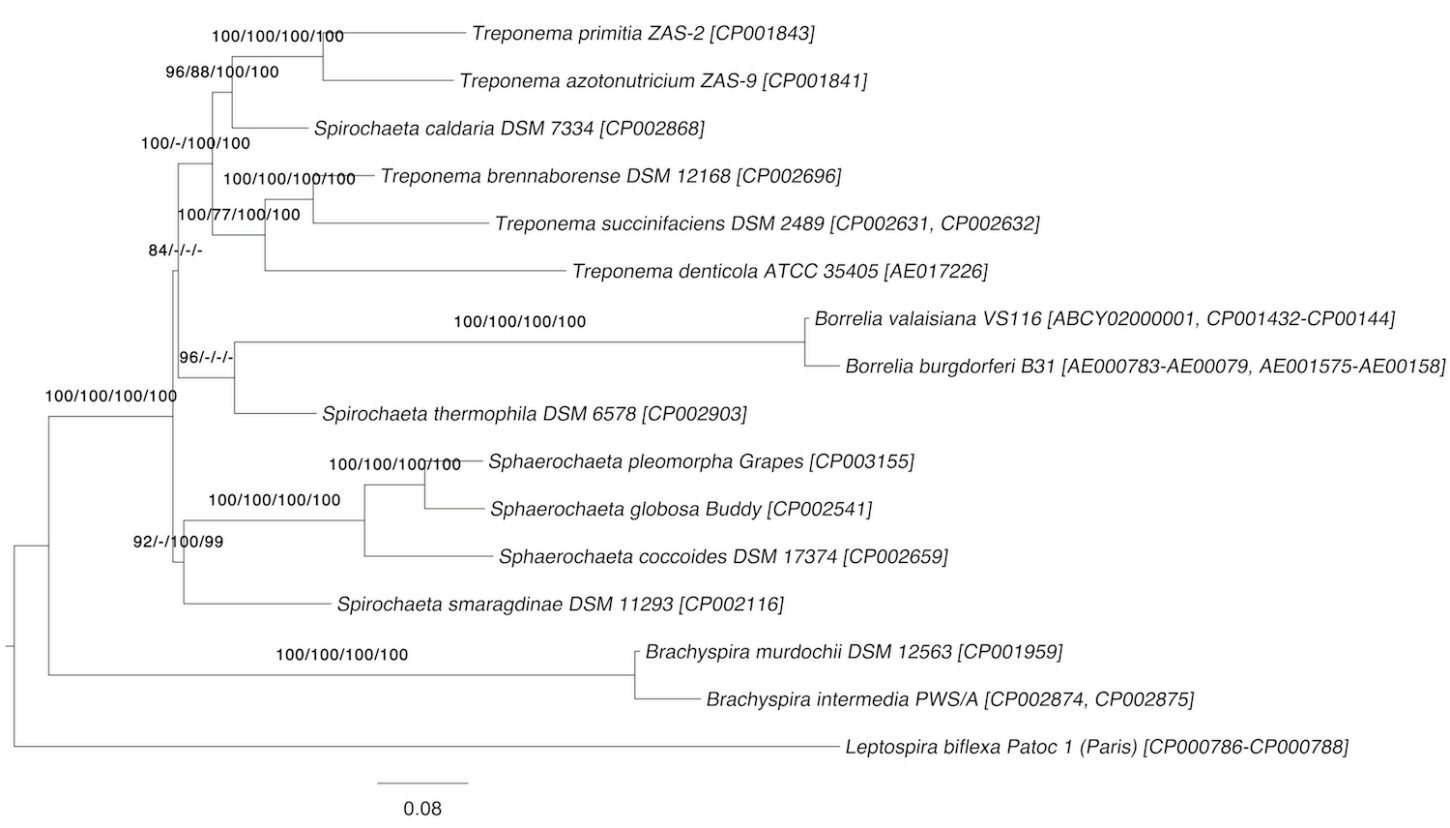
Phylogenetic tree inferred from completely sequenced genomes of the *Spirochaeta* type strains. The tree was inferred from 11,131 gene-content characters under the maximum likelihood (ML) criterion and rooted with *Leptospira*. The branches are scaled in terms of the expected number of substitutions per site. Numbers above the branches are bootstrapping support values (if larger than 60%) from (i) maximum-likelihood gene-content analysis; (ii) maximum-parsimony gene-content analysis; (iii) maximum-likelihood supermatrix analysis; (iv) maximum-parsimony supermatrix analysis. For further details see [19].

Amino-acid sequences from 16 *Spirochaetaceae* and outgroups (other *Spirochaetes* families) completed type-strain genomes were retrieved from INSDC and used in a phylogenomic analysis of the group, as described previously [19,61]. One of the previous taxonomic consequences for the genus *Spirochaeta* was the assignment of *S. coccoides* to the genus *Sphaerochaeta* [19]. Here the gene-content phylogeny from the previously conducted analyses is depicted together with the bootstrap support values from all four applied approaches (Fig. 6).

All phylogenomic methods support the sister-group relationship of *S. caldaria* and two *Treponema* species, *T. azotonutricium* and *T. primitia* (88-100%). These methods corroborate the results of the 16S rRNA analysis that *Treponema* is paraphyletic. It was previously concluded that taxonomic revisions were necessary [19]. Here we revisit the definitions of *Spirochaeta* and *Treponema* and formally propose a number of revisions and emendations to solve these problems.

### Phenotypic data and taxonomic interpretation

Table 6 presents an overview of key morphological and physiological features of *S. caldaria, S. zuelzerae and S. stenostrepta* compared with the genus descriptions of *Spirochaeta* and *Treponema*.

**Table 6:** Typical features of reference taxa compared to the three *Spirochaeta* species placed within *Treponema*.

	***Spirochaeta caldaria* [**1**]**	***Spirochaeta zuelzerae* [**62**]**	***Spirochaeta stenostrepta* [**63**]**	**Genus *Spirochaeta* [**31,34-36,55**]**	**Genus *Treponema* [**55,64**]**
**Cell shape**	helical	helical	helical	helical; spherical bodies under unfavorable growth conditions	helical; spherical bodies under unfavorable growth conditions
**Pathogenicity**	non pathogenic	non pathogenic	non pathogenic	non pathogenic	some species are pathogenic
**Biotic relationship**	free living	free living	free living	free living	primarily host-associated
**Size [µm]**	0.2-0.3 by 15-45	0.2-0.35 by 8-16	0.2-0.3 by 15-45	0.2-0.75 by 5-250	0.1-0.7 by 5-20
**Motility**	motile	motile	motile	motile	motile
**Flagellation**	flagella 1-2-1	flagella 1-2-1	2 periplasmic flagella	2 periplasmic flagella (exception: *S. plicatillis*, which has many flagella)	one or more periplasmic flagella
**Relationship to O_2_**	obligately anaerobe	obligately anaerobe	obligately anaerobe	obligately anaerobe or facultatively anaerobe	obligately anaerobe or microaerophilic
**Utilizes**	carbohydrates, no amino acids	carbohydrates	carbohydrates	a variety of carbohydrates, no amino acids	carbohydrates or amino acids
**Fermentation products**	acetate, lactate, CO_2_, H_2_	acetate, lactate, CO_2_, H_2_	acetate, ethanol, CO_2_, H_2,_ (lactate)	acetate, ethanol, CO_2_, H_2_	
**G+C content** **[mol%]**	46	56	60	51-65 [35] 44-65 [34]	37-54

The genus descriptions of *Spirochaeta* and *Treponema* evolved during the decades, and became less restrictive and differentiating. This makes a correct diagnosis of the genera within the family *Spirochaetaceae* difficult. In 2010, Leschine and Paster listed characteristics for the differentiation of the genus *Spirochaeta* from other genera of spirochetes [65]. In contrast to the genus *Treponema,* members of the genus *Spirochaeta* are free-living and cannot use amino acids as energy source. *S. caldaria*, *S. zuelzerae* and *S. stenostrepta* have both characteristics (Table 6), but based on 16S rRNA comparison these three *Spirochaeta* spp. are more closely related to species of *Treponema* [65]. The utilization of amino acids is not a restrictive criterion as some *Treponema* species also lack the ability to use amino acids as an energy source (*T. bryantii* [66], *T. parvum* [67], *T. pectinovorum* [68] and *T. porcinum* [69]). As a consequence of the existence of free-living species of *Spirochaeta*, which are more closely related to species of *Treponema,* Leschine and Paster suggest that “free-living“ vs. “host-associated” may not be a reliable taxonomic criterion to differentiate species of *Spirochaeta* and *Treponema* [65].

*Spirochaeta zuelzerae* was originally described by Veldkamp in 1960 [62] as “*Treponema zuelzerae”*. Based on existing classification key at the time [70], Veldkamp placed his spirochete, on the basis of its cell length to the *Spirochaetaceae* and in its serological similarity to the genus *Treponema*, into the family “*Treponemaceae”*. Canale-Parola *et al*. 1968 criticized the classification based on cell length, as the size can vary depending on the growth phase of the culture [25]. Because of the similarity between Veldkamp’s spirochete and other species of *Spirochaeta* Canale-Parola *et al*. (1968) suggested that *T. zuelzerae* should be included in the genus *Spirochaeta*, as *Spirochaeta zuelzerae*. Thus the name *S. zuelzerae* was revived and validly published [71].

Apparently the phenotypic definitions of both genera are vague and non-differential. The range of the features expressed as continuous numbers (cell size, GC content) numerically overlap, and the ranges of the other, discrete features logically overlap. Even the biotic relationships are expressed merely as a tendency, with *Treponema* assumed to be “primarily host-associated”; a criterion that has been questioned earlier [65]. *S. stenostrepta* and *S. zuelzerae* do not fit the description of *Treponema*, and only with regard to a single character, the GC content, which can hardly outweigh the phylogenetic evidence presented in Fig. 1, Fig. 6 and [19]. As far as this can be inferred from the distribution of relative COG counts (Fig. 4), genomic data make it unlikely that physiological characteristics can be found to differentiate between *Spirochaeta* and *Treponema*.

On the basis of the phylogenetic evidence presented above (Fig. 1, Fig. 6) and in [19], the reclassification of *S. caldaria*, *S. stenostrepta* and *S. zuelzerae* into the genus *Treponema* is proposed. This also makes emendation of the genus necessary, as the current description excludes a small number of features found in these three species. Our proposal is based on two principles, (i) that all taxa should be monophyletic (or, more precisely, no taxon should be demonstrably non-monophyletic) [39,42,72,73] and (ii) that as few taxonomic changes should be conducted as possible. The second principle rules out the alternative solution to merge both genera (which would then also make the inclusion of *Sphaerochaeta* and perhaps *Borrelia* necessary).

### Emended description of the genus *Treponema* Schaudinn 1905 emend. Smibert 1974 (Approved Lists 1980)

The description of the genus *Treponema* is the one given by Norris *et al*. [74], with the following modification.

The GC content is between 37 and 60 mol%. The biotic relationship is either host associated or free living.

### Description of *Treponema caldaria* (Pohlschroeder *et al*. 1994) Abt, Göker and Klenk, comb. nov.

Basonym: *Spirochaeta caldaria* Pohlschroeder *et al.* 1994.

The characteristics of the species are given in the species description by Pohlschroeder *et al*. 1994 [1].

The type strain is H1^T^ (= DSM 7334 = ATCC 51460).

### Description of *Treponema stenostrepta* (Zuelzer *et al*. 1912) Abt, Göker and Klenk, comb. nov.

Basonym: *Spirochaeta stenostrepta* (Zuelzer *et al*. 1912)

The characteristics of the species are given in the species description by Zuelzer *et al*. 1912 [63]. The type strain is Z1^T^ (= DSM 2028 = ATCC 25083).

### Description of *Treponema zuelzerae* (Canale-Parola 1980) Abt, Göker and Klenk, comb. nov.

Basonym: *Spirochaeta zuelzerae* (ex Veldkamp 1960) Canale-Parola 1980,

This species was originally described by Veldkamp 1960 as “*Treponema zuelzerae”* [62] but that name did not appear on the Approved Lists. The name was subsequently revived and validly published as *Spirochaeta zuelzerae* [25,71].

The characteristics of the species are given in the species description by Veldkamp 1960 [62]. The type strain is ATCC 19044 (= DSM 1903).
